# Retrospective analysis of patterns of opioid overdose and interventions delivered at a tertiary hospital emergency department: impact of COVID-19

**DOI:** 10.1186/s12873-022-00604-w

**Published:** 2022-04-09

**Authors:** Katherine L. Potaka, Rebecca Freeman, Danny Soo, Nam-Anh Nguyen, Tin Fei Sim, Joanna C. Moullin

**Affiliations:** 1 Curtin Medical School, Faculty of Health Sciences, Curtin University, Perth, WA Australia; 2 Sir Charles Gairdner Hospital, Perth, WA Australia

**Keywords:** Emergency department, Interventions, Opioid overdose, Take-home naloxone

## Abstract

**Background:**

Opioid-related overdoses cause substantial numbers of preventable deaths. Naloxone is an opioid antagonist available in take-home naloxone (THN) kits as a lifesaving measure for opioid overdose. As the emergency department (ED) is a primary point of contact for patients with high-risk opioid use, evidence-based recommendations from the Society of Hospital Pharmacists of Australia THN practice guidelines include the provision of THN, accompanied by psychosocial interventions. However, implementation of these guidelines in practice is unknown. This study investigated ED opioid-related overdose presentations, concordance of post-overdose interventions with the THN practice guidelines, and the impact, if any, of the SARS-CoV-2 (COVID-19) pandemic on case presentations.

**Methods:**

A single-centre retrospective audit was conducted at a major tertiary hospital of patients presenting with overdoses involving opioids and non-opioids between March to August 2019 and March to August 2020. Patient presentations and interventions delivered by the paramedics, ED and upon discharge from the ED were collated from medical records and analysed using descriptive statistics, chi square and independent T-tests.

**Results:**

The majority (66.2%) of patients presented to hospital with mixed drug overdoses involving opioids and non-opioids. Pharmaceutical opioids were implicated in a greater proportion (72.1%) of overdoses than illicit opioids. Fewer patients presented in March to August 2020 as compared with 2019 (26 vs. 42), and mixed drug overdoses were more frequent in 2020 than 2019 (80.8% vs. 57.1%). Referral to outpatient psychology (22.0%) and drug and alcohol services (20.3%) were amongst the most common post-discharge interventions. Naloxone was provided to 28 patients (41.2%) by the paramedics and/or ED. No patients received THN upon discharge.

**Conclusions:**

This study highlights opportunities to improve ED provision of THN and other interventions post-opioid overdose. Large-scale multi-centre studies are required to ascertain the capacity of EDs to provide THN and the impact of COVID-19 on opioid overdose presentations.

**Supplementary Information:**

The online version contains supplementary material available at 10.1186/s12873-022-00604-w.

## Background

Opioid overdose is recognised globally as a significant public health problem [1]. Use of pharmaceutical and non-pharmaceutical opioids, for example codeine, fentanyl and heroin, cause considerable individual and societal burden [2]. A 2020 Australian report revealed 75% of drug-induced deaths were unintentional [2]. Opioids were identified as the leading cause of unintentional drug-induced death in Australia, with the number involving opioids increasing by 66% from 2006 to 2018 [2]. In the 2015–2016 financial year, heroin use and misuse of pharmaceutical opioids cost Australia $5.6 billion dollars in tangible costs, and a further $10.1 billion in intangible costs due to premature deaths [3]. Internationally, 110,000 (67%) of the 167,000 deaths due to substance use disorders (SUDs) in 2017 were attributed to opioids [1]. We are facing an “opioid epidemic” [1].

The opioid epidemic has been compounded by the SARS-CoV-2 (COVID-19) pandemic [4–9]. Compelling evidence is emerging to suggest a rise in opioid overdoses during the COVID-19 pandemic, possibly due to the accompanying social isolation, financial stress and healthcare service disruptions [4, 7]. Holland et al. found a 32% increase in the number of opioid overdose presentations to United States (US) emergency departments (EDs) during 2020 as compared with 2019 [7]. Western Australia underwent its harshest lockdown period in March and April 2020 with social gatherings limited to 2 persons only [10]. Varying restrictions have been enforced by the Western Australian government for international and interstate travel since the onset of the pandemic, from mandatory 14-day hotel or at-home quarantine to complete border closures [10]. It is predicted that these travel restrictions and limitations on social interactions have impacted drug supply and drug use behaviours [8].

Survivors of non-fatal overdose are at high risk of subsequent overdose [11–13]. As such, interventions delivered in the ED post-overdose offer a unique opportunity to tackle the opioid epidemic [14, 15]. Naloxone, an opioid antagonist, is a life-saving treatment for opioid overdose that has traditionally been given by paramedics and in the ED [16, 17]. However, naloxone is now available in dosage forms suitable for use by laypersons who witness an overdose [16, 18]. Under the Australian take-home naloxone (THN) pilot, THN kits are accessible free-of-charge to high-risk patients and their families, friends, or anyone who is likely to witness an opioid overdose [19]. The pilot program began in December 2019 and is managed in Western Australia by the Mental Health Commission [20]. Approximately 25% of patients who are trained and supplied with THN will go on to use it within 1 year to reverse an overdose [18]. Yet, the distribution of THN is limited in many ED settings [21–23], despite World Health Organization (WHO) support and studies consistently demonstrating its positive impact [16, 18, 24]. In accordance with the Society of Hospital Pharmacists of Australia practice guidelines on THN in Australian hospitals, ED patients with opioid toxicity should receive THN in conjunction with counselling about its use [25]. THN should be provided alongside psychosocial interventions, such as psychiatric assessment, peer-recovery coach programs and social services referral, to prevent recurrent overdose [16, 26]. Since this Society of Hospital Pharmacists of Australia guideline was released in November 2020, there is currently no information about its implementation in practice.

Studies investigating the capacity to which these potentially life-saving interventions are delivered post-overdose are limited. Information regarding opioid overdose presentation numbers, and the delivery of post-discharge interventions, is required to provide an indication of the capacity of Australian EDs to meet the WHO recommendations and Society of Hospital Pharmacists of Australia guidelines. As the ED is a primary, and perhaps only, point of contact with the healthcare system for patients living with SUDs, further understanding of this topic is critical to address the upward shift in opioid-induced mortality. Furthermore, to date, changes in opioid overdose presentations with the onset of the COVID-19 pandemic have not been evaluated in Australia.

## Methods

### Aim

This study aimed to assess ED overdose presentations involving opioids and how they align with current Society of Hospital Pharmacists of Australia practice guidelines for THN in Australian hospitals. Specifically, the study aimed to determine: (1) the frequency of overdose presentations that involved pharmaceutical and non-pharmaceutical opioids, (2) the characteristics and comorbidities of opioid overdose patients and their ED presentations, (3) the interventions and post-discharge management strategies provided after opioid overdose presentations and (4) differences in opioid overdose presentations before and during the COVID-19 pandemic.

### Study design

A single-centre, retrospective observational audit was conducted at a tertiary hospital in Australia, using data obtained from medical records. Governance Evidence Knowledge Outcome (GEKO) (Quality Activity number 39844; approved 26 February 2021) and reciprocal Curtin human research ethics (HRE2021-0095) approval were obtained prior to data collection.

### Participants and sampling

Patients 18 years and older admitted to the ED and diagnosed with opioid-related overdose from March to August 2019 (pre-COVID-19) and March to August 2020 (COVID-19) were included. Opioid-related overdoses were defined as overdoses where opioids were the sole contributor or contributed in combination with other drugs (mixed drug overdoses). Patients presenting with overdose are admitted under the toxicology team for immediate review, investigations and treatment.

A list of eligible cases was obtained from the Medical Records Department using the Australian ICD-10-AM clinical codes: EKB00 (drug and alcohol), X42 (accidental poisoning by and exposure to narcotics and hallucinogens) and X62 (intentional self-poisoning by and exposure to narcotics and hallucinogens). Patient numbers for each ICD-10-AM code were tabulated (Supplementary Table 1). Following this, ICD-10-AM codes indicative of opioid-related overdose were identified: T40.1 (heroin), T40.6 (other and unspecified narcotics), T40.2 (other opioids), X42 and X62. These codes were used to shortlist appropriate patients and data were collated from corresponding files where the inclusion criteria were met.

### Data collection

Two auditors extracted data from patients’ medical records. To ensure accuracy and robustness in the data collected, the auditors attended a one-week ED visit with the toxicology team to familiarise themselves with procedures and documentation in relation to overdose presentations prior to data collection. To further improve data accuracy, the first 10% of patients were reviewed by both auditors and cross-checked. Disparities were evaluated and resolved prior to continuation of the audit.

Data transcribed from medical records comprised of demographic information, current medications and comorbidities, overdose factors (e.g. type of opioids used and their Standard for the Uniform Scheduling of Medicines and Poisons (SUSMP) Schedule), ED presentation characteristics (e.g. date of admission, length of stay and arrival mode) and interventions delivered after medical assistance arrived (pre-ED), in the ED and upon discharge from the ED. Pharmaceutical opioids were defined as those classified under the SUSMP as Schedule 2, 3, 4 and 8, while non-pharmaceutical opioids were classified as Schedule 9 [27].

### Data analysis

Data were analysed using SPSS version 27. Continuous and categorical variables were analysed using descriptive statistics. Inferential statistics (chi square tests and independent T-tests) were used to explore associations between patient characteristics, their presentations and the interventions provided, and to detect differences in overdose presentations between the audit periods in 2019 and 2020.

## Results

### Description of sample

A total of 108 medical records of patients presenting during the audit period with a diagnosis indicative of opioid overdose were identified. Of these, 42 were either not overdoses (e.g. adverse drug reaction) or were not opioid-related overdoses (e.g. cannabis overdose) and, therefore, were excluded. Data were extracted from 66 patients who presented with opioid-related overdose. One patient presented 3 times during the audit period and, therefore, 68 cases were included in the primary analysis. There was 32 male and 36 female cases included and the mean age of patients at presentation was 40.6 ± 16.8 years (Table 1).

**Table 1:** Summary statistics of patients presenting for opioid overdose during March to August 2019 and 2020

Variable	Total n (%) *N* = 68	March-to-August n (%)		*P*-value
		**2019** ***N* = 42**	**2020** ***N* = 26**	
Gender				
Male	32 (47.1)	17 (40.5)	15 (57.7)	0.258
Female	36 (52.9)	25 (59.5)	11 (42.3)	
Age (years) (mean ± SD)	40.6 ± 16.8	38.7 ± 16.2	43.6 ± 17.6	0.246
Arrival mode				
Ambulance	55 (80.9)	37 (88.1)	18 (69.2)	0.108
Other	13 (19.1)	5 (11.9)	8 (30.8)	
Nature of overdose^a^				
Intentional	32 (47.8)	22 (53.7)	10 (38.5)	0.336
Unintentional	35 (52.2)	19 (46.3)	16 (61.5)	
Opioids used in overdose^b^				
Pharmaceutical	49 (72.1)	32 (76.2)	17 (65.4)	0.492
Non-pharmaceutical	19 (27.9)	10 (23.8)	9 (34.6)	
Drugs used in overdose				
Opioids only	23 (33.8)	18 (42.9)	5 (19.2)	0.082
Opioids and non	45 (66.2)	24 (57.1)	21 (80.8)	
opioids				
Number of drugs consumed in overdose				
1–3	49 (72.1)	34 (81.0)	15 (57.7)	0.072
4–7	19 (27.9)	8 (19.0)	11 (42.3)	
Inpatient admission				
Yes	18 (26.5)	11 (26.2)	7 (26.9)	1.000
No	50 (73.5)	31 (73.8)	19 (73.1)	
ED and/or ambulance naloxone provided				
Yes	28 (41.2)	14 (33.3)	14 (53.8)	0.157
No	40 (58.8)	28 (66.7)	12 (46.2)	

*ED* emergency department, *SD* standard deviation

^a^Excluding 1 deceased patient for whom the nature of overdose was not determined

^b^Pharmaceutical opioids and opioid derivatives are classified under the Standard for the Uniform Scheduling of Medicines and Poisons (SUSMP) as Schedule 2, 3, 4 and 8, while non-pharmaceutical opioids are classified as Schedule 9

### Emergency department presentation

Of the 68 cases reviewed, 42 (61.8%) presented in 2019 and 26 (38.2%) presented during the 2020 6-month audit period of March to August (Table 1). The majority of patients arrived by ambulance (*N* = 55, 80.9%), while the remaining arrived via private transport (*N* = 10, 14.7%), hospital transfer (*N* = 2, 2.9%) or were brought in by police (*N* = 1, 1.5%). There was a non-significant trend of a reduction in arrival by ambulance during the COVID-period of 2020 (88.1% vs. 69.2%). The median length of stay was 7 h (IQR: 9). Eighteen patients (26.5%) were admitted as an inpatient and, of these, the majority (*N* = 11, 61.1%) were admitted to the intensive care unit. Half of the inpatients returned from the intensive care unit to the ED prior to discharge. This is a common practice within the hospital whereby patients are moved to an observation ward within the ED to await discharge. Aspiration pneumonia was reported for 7 of the 18 patients (38.9%) as the primary reason for hospital admission. One patient died in hospital as a consequence of their overdose.

### Medical history

A history of SUDs, such as opioid, benzodiazepine and alcohol dependence, was evident in 55.9% of cases (*N* = 38). A total of 23.5% (*N* = 16) of patients presented previously to the hospital’s ED for unspecified drug overdose, and 11.8% (*N* = 8) for opioid-related overdose.

Men were more likely to report drug-related comorbidities than females (71.9% vs. 41.7%; *p* < *0.05*) (Table 2). Excluding SUDs, the most common comorbidity was mental, behavioural or neurodevelopmental disorders (ICD-11 code 06) (Table 2), with 61.8% (*N* = 42) of patients affected overall, 48.5% (*N* = 33) presenting with comorbid depression and 38.2% (*N* = 26) presenting with anxiety.

**Table 2:** Frequency of patient comorbidities amongst 68 opioid-related overdose cases presenting to the ED

ICD-11-AM Clinical Code	Frequency (%)
06-Mental, behavioural or neurodevelopmental disorders^a^	42 (61.8)
11-Diseases of the circulatory system	14 (20.6)
12-Diseases of the respiratory system	14 (20.6)
08-Diseases of the nervous system	13 (19.1)
01-Certain infectious or parasitic diseases	12 (17.6)
05-Endocrine, nutritional or metabolic disorders	11 (16.2)
21-Symptoms, signs or clinical findings, not elsewhere classified	10 (14.7)
24-Factors influencing health status or contact with health services	9 (13.2)
13-Diseases of the digestive system	8 (11.8)
15-Diseases of the musculoskeletal system	7 (10.3)
22-Injury, poisoning or certain other consequences of external causes	6 (8.8)
02-Neoplasms	6 (8.8)
16-Diseases of the genitourinary system	6 (8.8)
07-Sleep–wake disorders	5 (7.4)
03-Diseases of the blood or blood forming organs	2 (2.9)
17-Conditions related to sexual health	2 (2.9)
04-Diseases of the immune system	1 (1.5)
09-Diseases of the visual system	1 (1.5)
14-Diseases of the skin	1 (1.5)

*ICD* International Classification of Diseases, *ED* emergency department

^a^Excluding substance use disorders

Amongst the 68 cases, 25 (36.8%) reported taking 5 or more current medications. Opioids were listed as a current medication in 28 (41.2%) cases and, overall, tramadol was the most common (*N* = 16, 23.5%). Prescribing indications for opioids included chronic back pain, fibromyalgia, ankylosing spondylitis, recent trauma, cancer pain and post-surgical pain management. Psychotropic drugs were reported as a current medication in 67.6% (*N* = 46) of cases. Of these, pregabalin was the most common (*N* = 16, 23.5%), followed by diazepam (*N* = 7, 10.3%), and quetiapine (*N* = 7, 10.3%). Benzodiazepines were listed as a current medication in 23.5% of cases (*N* = 16).

### Overdose factors

Of the 68 cases, tramadol was the most common drug that contributed to overdose (*N* = 21, 30.9%), followed by heroin (*N* = 19, 27.9%) and codeine (*N* = 15, 22.1%) (Table 3). Males were more than twice as likely to overdose on heroin than females (40.6% vs. 16.7%). The majority of heroin overdoses were single drug overdoses (*N* = 14, 73.7%) and were unintentional (*N* = 16, 84.2%). In 49% (*N* = 24) of pharmaceutical opioid overdoses, the opioid was prescribed for the patient. In the remaining cases, opioids were sourced from family or friends (*N* = 4, 8.2%), illicitly (*N* = 2, 4.1%) or the source was not documented (*N* = 19, 38.8%).

**Table 3:** Frequency of opioids used in opioid-only and mixed drug overdoses

**Opioid Only Overdoses (*N*** **= 23, 33.8%)**	
**Opioid used**	**Frequency** ***N* (%)**
Heroin	14 (60.9)
Tramadol	4 (17.4)
Oxycodone	4 (17.4)
Buprenorphine (patch)	1 (4.3)
Poppy seed tea^a^	1 (4.3)
**Mixed Drug Overdoses (*N* = 45, 66.2%)**	
**Opioid used**	**Frequency** ***N* (%)**
Tramadol	17 (37.8)
Codeine	15 (33.3)
Oxycodone	6 (13.3)
Tapentadol	5 (11.1)
Heroin	5 (11.1)
Buprenorphine (sublingual)	4 (8.9)
Methadone (intravenous)	1 (2.2)
Methadone (oral)	1 (2.2)
Hydromorphone	1 (2.2)

^a^Poppy seed tea contains a mixture of morphine, codeine, papaverine and thebaine and, in sufficient quantities, produces psychoactive effects

The majority of cases were mixed overdoses involving opioid and non-opioid drugs (*N* = 45, 66.2%). Intentional overdoses were significantly more likely to involve pharmaceutical opioids (93.8% vs. 54.3%; *p* < *0.001*) and were reported significantly more frequently as mixed drug overdoses compared with unintentional overdoses (84.4% vs. 51.4%; *p* < *0.05*) (Table 4). Paracetamol (*N* = 16, 35.6%), alcohol (*N* = 13, 28.9%), pregabalin (*N* = 12, 26.7), diazepam (*N* = 6, 13.3%) and clonazepam (*N* = 6, 13.3%) were the most commonly used drugs alongside opioids. Benzodiazepines were implicated in 37.8% (*N* = 17) of mixed drug overdoses. In 14 of the 45 cases involving non-opioids (31.1%) a combination of paracetamol, pregabalin and/or quetiapine with a benzodiazepine was consumed. Other drug types commonly used included antidepressants (*N* = 10, 22.2%), hallucinogens (*N* = 6, 13.3%) and stimulants (*N* = 5, 11.1%).

**Table 4:** Summary statistics for patients presenting to the ED with intentional or unintentional opioid-related overdose^a^

Variables	Intentional Overdoses *N* (%) (*N* = 32)	Unintentional overdoses *N* (%) (*N* = 35)	*P*-value
Gender			
Male	9 (28.1)	23 (65.7)	0.005
Female	23 (71.9)	12 (34.3)	
Age (years) (mean ± SD)	37.9 ± 15.7	42.9 ± 17.8	0.225
Mental, behavioural or neuro-developmental disorders (ICD-11 code 06)^b^			
Yes	24 (75.0)	18 (51.4)	0.049
No	8 (25.0)	17 (48.6)	
Substance use disorders			
Yes	11 (34.4)	26 (74.3)	0.002
No	21 (65.6)	9 (25.7)	
Length of stay (mean ± SD)	15.2 ± 17.8	8.3 ± 5.2	0.042
Arrival mode			
Ambulance	24 (75.0)	30 (85.7)	0.425
Other	8 (25.0)	5 (14.3)	
Opioids used in overdose^c^			
Pharmaceutical	30 (93.8)	19 (54.3)	< 0.001
Non-pharmaceutical	2 (6.3)	16 (45.7)	
Drugs used in overdose			
Opioids only	5 (15.6)	17 (48.6)	0.009
Opioids and non-opioids	27 (84.4)	18 (51.4)	
If pharmaceutical opioids consumed, prescribed for			
Self	15 (50)	9 (47.4)	1.000
Other/Illicit source/Unknown	15 (50)	10 (52.6)	
Number of drugs consumed in overdose			
1–3	21 (65.6)	27 (77.1)	0.439
4–7	11 (34.4)	8 (22.9)	
Inpatient admission			
Yes	8 (25.0)	9 (25.7)	1.000
No	24 (75.0)	26 (74.3)	
ED and/or ambulance naloxone provided			
Yes	4 (12.5)	24 (68.6)	< 0.001
No	28 (87.5)	11 (31.4)	
Drug and alcohol assessment provided or offered but declined by patient			
Yes	5 (15.6)	13 (37.1)	0.087
No	27 (84.4)	22 (62.9)	
Engagement with drug and alcohol services documented in post-discharge management plan or suggested but declined by patient			
Yes	4 (14.3)	12 (38.7)	0.070
No	24 (85.7)	19 (61.3)	
Follow-up psychology (including MHCP), counselling or psychiatry (including transfer to psychiatric hospital) documented in post-discharge management plan			
Yes	17 (60.7)	6 (19.4)	0.003
No	11 (39.3)	25 (80.6)	

*ED* emergency department, *SD* standard deviation, *MHCP* mental health care plan

^a^Excluding 1 deceased patient for whom the nature of overdose was not determined

^b^Excluding substance use disorders

^c^Pharmaceutical opioids and opioid derivatives are classified under the Standard for the Uniform Scheduling of Medicines and Poisons (SUSMP) as Schedule 2, 3, 4 and 8, while non-pharmaceutical opioids are classified as Schedule 9

### Interventions delivered

Of the 55 patients that arrived by ambulance, 9 (16.4%) received naloxone administered by paramedics (Table 5). One patient received naloxone delivered intranasally by a bystander prior to arrival of the paramedics. Within the ED, naloxone was delivered to 33.8% (*N* = 23) of patients. The median total dose of naloxone provided within the ambulance and ED was 550 µg, though there was significant variation from 100 µg to 14,212 µg. Assessments by the drug and alcohol service were provided to 20.6% (*N* = 14) of patients. A further 4 patients declined, 14 were admitted as inpatients, 1 patient was assessed on a recent admission and 1 was discharged against medical advice. Of the patients who were discharged from the ED, no patients received THN. A recommendation for patients to follow up with their general practitioner was most frequently documented within the post-discharge management plan (*N* = 23, 39.0%), followed by psychiatry (*N* = 13, 22.0%) and indirect referral or continued engagement with drug and alcohol services (*N* = 12, 20.3%). For patients with SUDs, drug and alcohol service engagement was a more common post-discharge intervention in 2020 as compared with 2019 (56.3% vs. 26.3%).

**Table 5:** Interventions commonly delivered following opioid-related overdoses in the ambulance, tertiary hospital ED and upon discharge

Interventions Delivered	March-to-August *N* (%)		
	**2019**	**2020**	**Total**
**Ambulance Interventions**	***N* = 37**	***N* = 18**	***N* = 55**
Oxygen	13 (35.1)	4 (22.2)	17 (30.9)
Oropharyngeal airway insertion	10 (27.0)	2 (11.1)	12 (21.8)
IV/IM naloxone	9 (24.3)	0 (0)	9 (16.4)
Declined IV/IM naloxone	0 (0)	1 (5.6)	1 (1.8)
**ED Interventions**	***N* = 42**	***N* = 26**	***N* = 68**
IV/IM naloxone	8 (19.0)	14 (53.8)	22 (32.4)
Psychiatric review	12 (28.6)	6 (23.1)	18 (26.5)
Declined psychiatric review	3 (7.1)	0 (0)	3 (4.4)
Paracetamol	9 (21.4)	6 (23.1)	15 (22.1)
Drug and alcohol assessment	6 (14.3)	8 (30.8)	14 (20.6)
Declined drug and alcohol assessment	2 (4.8)	2 (7.7)	4 (5.9)
Oral/IV benzodiazepines (diazepam, midazolam, lorazepam and/or temazepam)	10 (23.8)	4 (15.4)	14 (20.6)
Social work team review	7 (16.7)	3 (11.5)	10 (14.7)
Declined social work team review	1 (2.4)	1 (3.8)	2 (2.9)
Intubation and ventilation	6 (14.3)	3 (11.5)	9 (13.2)
Naloxone infusion	2 (4.8)	5 (19.2)	7 (10.3)
Self-harm and crisis counselling services team review	3 (7.1)	2 (7.7)	5 (7.4)
N-acetylcysteine infusion	2 (4.8)	3 (11.5)	5 (7.4)
**Post-discharge Interventions**	***N* = 35**	***N* = 24**	***N* = 59**^**a**^
General practitioner follow-up	15 (42.9)	8 (33.3)	23 (39.0)
Psychology	10 (28.6)	3 (12.5)	13 (22.0)
Indirect referral or continued engagement with community, non-government organisation or private drug and alcohol services	5 (14.3)	7 (29.2)	12 (20.3)
Declined drug and alcohol services engagement	2 (5.7)	2 (8.3)	4 (6.8)
Psychiatrist follow-up or transfer/referral to psychiatric hospital	6 (17.1)	5 (20.8)	11 (18.6)
Changes to opioid and/or psychotropic medications (ceased, reduced dose and/or modified patient access)	5 (14.3)	2 (8.3)	7 (11.9)
Self-help and crisis counselling services information and/or follow-up	4 (11.4)	2 (8.3)	6 (10.2)
Harm minimisation education, including education regarding risks of overdose	4 (11.4)	1 (4.2)	5 (8.5)
Mental health crisis information and contact numbers (e.g., Mental Health Emergency Response Line number)	4 (11.4)	1 (4.2)	5 (8.5)
Mental health care plan (to be provided by general practitioner)	4 (11.4)	0 (0)	4 (6.8)
Family involvement (e.g., psychoeducation or facilitation of discharge management plan)	1 (2.9)	3 (12.5)	4 (6.8)

*ED* emergency department, *IV* intravenous, *IM* intramuscular, *NSAID* non-steroidal anti-inflammatory drug

^a^Nine patients were admitted as inpatients and not discharged from the ED

## Discussion

This study observed a greater proportion of opioid-related overdoses involving pharmaceutical opioids as compared to heroin. Mixed drug overdoses involving non-opioids were more frequent than opioid only overdoses. This finding was more pronounced during March to August 2020 as compared to the same period in 2019 (pre-COVID-19), and for those presenting with intentional overdoses. A reduction in the number of patients presenting with opioid-related overdoses between 2019 and 2020 was evident, which may be attributed to the COVID-19 pandemic. Mental health comorbidities were prominent amongst the patient population, especially depression and anxiety. As part of the post-discharge plan for patients presenting with opioid-related overdose, several strategies were documented including psychiatry follow-up and drug and alcohol services referral. However, no patients received THN. Barriers to the provision of THN in hospital EDs need to be addressed to complement and support wider efforts to promote THN access and prevent opioid-induced mortality.

To our knowledge, this is the first study examining the provision of THN and other interventions post-opioid overdose in Australian EDs. Providing and educating people on THN is an effective strategy to reverse opioid overdose and prevent mortality [16]. The 2020 Society of Hospital Pharmacists of Australia guidelines state that THN should be provided to patients presenting with opioid toxicity, those who inject opioids, use opioid substitution therapy or are prescribed opioids for chronic pain [25]. While THN is available free-of-charge from 225 community drug and alcohol services and pharmacies in the Australian state of Western Australia under the THN pilot, only 1 hospital is registered with this program and 2 other hospitals are supplying THN to ED patients according to anecdotal reports [28]. Equity of access and care needs to be addressed, as no hospitals outside of the inner city are currently providing THN. Internationally, the underutilisation of THN programs is attributed to multiple barriers, including lack of time, training and institutional support [21, 29]. Furthermore, some healthcare workers hold stigmatising and inaccurate assumptions about naloxone distribution [30, 31]. There is a need to improve understanding and shift attitudes regarding THN to ensure survivors of opioid overdose, and those at risk of overdose from pharmaceutical and non-pharmaceutical drug use, have access to this life-saving intervention from the ED [25].

An expected finding of this study was the significant proportion of patients with comorbid mental health conditions. This was evident for those who intentionally overdosed (75%), as well as those who unintentionally overdosed (51.4%), which reflects a well-established relationship between SUDs and mental health conditions [32–34]. Those who intentionally overdosed were less likely to be given naloxone than those who unintentionally overdosed (*p* < 0.001). This may indicate intentional overdoses were less severe or opioids contributed to a lesser extent. Consistent with the latter, those who intentionally overdosed were significantly more likely to present with mixed drug overdoses (*p* < 0.05). Psychiatric team follow-up was more commonly documented in the post-discharge plan of those who intentionally overdosed than those who unintentionally overdosed (*p* < *0.05*). However, referral rates could be improved. Addressing the circumstances surrounding patients’ intentional overdoses and implementing treatment strategies is crucial to preventing self-harm [35].

A significant number of patients were referred to outpatient drug and alcohol services. However, outpatient referrals often indicated the provision of contact numbers and relied on patients to organise appointments. This is inconsistent with an ideal continuity of care approach where, for example, a clinical handover is conducted between healthcare providers with patient involvement or peer recovery coaches conduct motivational interviewing and facilitate referrals [15, 36]. A complicating factor with regards to organising referrals may be patients’ lack of motivation to change their drug use behaviours [37]. Requesting discharge and declining services was a common occurrence amongst the patient population, which may demotivate healthcare professionals to encourage patient change. The drug and alcohol team (at this hospital) extended their hours of operation considerably from 2019 to 2020, which was accompanied by a 30% increase in referral rates. Positive changes such as this are required to address the disconnect between EDs and community drug and alcohol services and optimise patient engagement in outpatient services following ED overdose presentations.

Pharmaceutical opioids were implicated in the majority of opioid-related overdoses included in this audit (72.1%). Comparatively, the Australian Institute of Health and Welfare reported the annual hospitalisation rate for overdoses involving pharmaceutical opioids was more than double that of overdoses involving heroin or opium (9.1 vs. 3.4 per 100,000 population) [38]. This is consistent with US trends, where in 2018 9.9 million people reported past-year use of prescription opioids for non-medical purposes and 800,000 reported heroin use [1]. In the current audit, pharmaceutical opioids prescribed for the patient were implicated in a substantial number of cases (*N* = 24). In recognition of the harm attributed to pharmaceutical opioids, the Therapeutic Goods Administration of Australia has established the Opioid Regulatory Advisory Group to alter opioid prescribing and dispensing practices [39]. For example, smaller pack sizes for immediate-release prescription opioid medications have become available to allow dispensing of the quantity required for the patient. This prevents surplus opioids circulating within the community that may cause inadvertent or deliberate harm [39]. Continual review of opioid use is required to lessen the burden of opioid-related harm.

A significant reduction in the number of ED presentations for opioid-related overdose was seen between 2019 and 2020 (*N* = 42 vs. 26, respectively). This finding correlates with anecdotal reports from healthcare workers at the hospital of fewer overdose presentations with the COVID-19 pandemic onset. In contrast, US studies have observed an increase in opioid overdose presentations from March 2020 [4, 7]. There are a number of possible explanations for the decline observed in this audit including (1) changes in drug access with border closures, (2) comparatively fewer well-meaning bystanders available to phone ambulances during isolation periods imposed in Australia, (3) increased fatalities (4) community expansion of the Australian THN pilot [20], and (4) a small sample size. To support the second of these hypotheses, patients were more likely to present by means other than ambulance transport during 2020 than 2019 (30.8% vs. 11.9%). While there was no significant difference in the proportion of overdoses involving pharmaceutical and non-pharmaceutical opioids, mixed drug overdoses involving non-opioids were more frequent in 2020 than 2019 (80.8% vs. 57.1%), as well as overdoses involving between 4 and 7 drugs (42.3% vs. 19.0%). These trends provide preliminary evidence to suggest a shift in drug use behaviours during 2020 as compared with 2019.

The findings of this study are subject to a number of limitations. As a small sample size, single centre study was conducted the outcomes cannot be generalised to other hospital EDs. However, results regarding characteristics of overdose presentations are consistent with current literature.^1,38^ Selecting the patient population using ICD-10-AM codes may have excluded eligible patients, but we included a large number of codes to capture the majority of presentations. Data outlining changes in overall ED presentations with COVID-19 was not obtained. If a decline in ED visits for all diagnoses was observed, this would limit the conclusions drawn regarding a reduction in opioid overdose presentations with the onset of COVID-19. Although, the results provide preliminary evidence of a decline in ED opioid overdose presentations with COVID-19 that warrants further investigation. This retrospective audit relied on documentation of patient characteristics, their overdose presentations and interventions delivered, which may be inaccurate or incomplete. For example, patients may not have been forthcoming with their comorbidities or healthcare workers may not have documented if they provided harm-minimisation education. To minimise the possibility of incomplete data we reviewed all available sources of information. Multi-centre, large-scale studies are required to further investigate ED opioid overdose presentations and the subsequent delivery of interventions.

## Conclusions

Consistent with previous research, overdoses involving pharmaceutical rather than non-pharmaceutical opioids accounted for the majority of presentations. A significant decline in the number of overdose presentations was observed with the onset of the COVID-19 pandemic, during which time patients were more likely to present with mixed drug overdoses involving opioids and non-opioids. Whilst referrals to psychiatry and outpatient drug and alcohol services were amongst the more common post-discharge interventions delivered, the frequency of this strategy could be improved along with continuity of care between the ED and outpatient services. Despite extensive evidence for THN use and increasing government support, no patients received THN. Survivors of non-fatal overdose are at exceptionally high risk of subsequent overdose and, thus, the ED is a crucial setting to implement harm reduction strategies. This study highlights opportunities to improve delivery of post-opioid overdose interventions as per current practice guidelines to address opioid-induced mortality.

## Supplementary Information

**Additional file 1:** **Supplementary Table 1.** Patients presenting to a tertiary hospital emergency department with specific diagnoses during 2019 and 2020.

## Data Availability

The datasets generated during the current study are not publicly available as they were commissioned by a third party but are available from the corresponding author on reasonable request.

## Abbreviations

SUD: Substance Use Disorder
US: United States
ED: Emergency Department
THN: Take-home naloxone
WHO: World Health Organization
GEKO: Governance Evidence Knowledge Outcome
