# Supplementary material for: Retrospective analysis of patterns of opioid overdose and interventions delivered at a tertiary hospital emergency department: impact of COVID-19
Source: BMC Emerg Med. 2022 Apr 9;22:62. doi: 10.1186/s12873-022-00604-w (PMC8994187; doi:10.1186/s12873-022-00604-w)
Supplement: Supplementary file 1 — Additional file 1: Supplementary Table 1. Patients presenting to a tertiary hospital emergency department with specific diagnoses during 2019 and 2020. [file 12873_2022_604_MOESM1_ESM.docx]

**Supplementary Table 1: Patients presenting to a tertiary hospital emergency department with specific diagnoses during 2019 and 2020.**

| ICD-10-AM Clinical Code | Diagnosis | March-to-August | | |
| --- | --- | --- | --- | --- |
|  |  | **2019**  ***N*= 513** | **2020**  ***N*= 492** | Total  *N*= 1005 |
|  | **Opioid** |  | | |
| X42 | Accidental poisoning by and exposure to narcotics and psychodysleptics [hallucinogens], not elsewhere classified | 31 | 31 | 62 |
| T40.1 | Heroin | 5 | 3 | 8 |
| X62 | Intentional self-poisoning by and exposure to narcotics and psychodysleptics [hallucinogens], not elsewhere classified | 29 | 19 | 48 |
| T40.6 | Other and unspecified narcotics | 3 | 2 | 5 |
| T40.2 | Other Opioids | 2 | 0 | 2 |
|  | **Non-opioids** |  | | |
| T39.1 | 4-Aminophenol derivatives | 5 | 8 | 13 |
| X41 | Accidental poisoning by and exposure to antiepileptic, sedative-hypnotic, antiparkinsonism and psychotropic drugs, not elsewhere classified | 44 | 45 | 89 |
| X40 | Accidental poisoning by and exposure to nonopioid analgesics, antipyretics and antirheumatics | 21 | 13 | 34 |
| T45 | Antiallergic and antiemetic drugs | 2 | 1 | 3 |
| T42.4 | Benzodiazepines | 17 | 13 | 30 |
| T50.2 | Carbonic-anhydrase inhibitors, benzothiadiazides and other diuretics | 2 | 0 | 2 |
| T40.5 | Cocaine | 1 | 0 | 1 |
| T38.3 | Insulin and oral hypoglycaemic [antidiabetic] drugs | 1 | 2 | 4 |
| X61 | Intentional self-poisoning by and exposure to antiepileptic, sedative-hypnotic, antiparkinsonism and psychotropic drugs, not elsewhere classified | 130 | 138 | 268 |
| X60 | Intentional self-poisoning by and exposure to nonopioid analgesics, antipyretics and antirheumatics | 45 | 47 | 92 |
| F10.0 | Mental and behavioural disorders due to use of alcohol, acute intoxication | 3 | 1 | 4 |
| T43.2 | Other and unspecified antidepressants | 10 | 5 | 15 |
| T43.5 | Other and unspecified antipsychotics and neuroleptics | 13 | 13 | 26 |
| T40.9 | Other and unspecified psychodysleptics [hallucinogens] | 11 | 11 | 22 |
| T46.2 | Other antidysrhythmic drugs, not elsewhere classified | 0 | 1 | 1 |
| T42.6 | Other antiepileptic and sedative-hypnotic drugs | 3 | 2 | 5 |
| T46.5 | Other antihypertensive drugs, not elsewhere classified | 5 | 0 | 5 |
| T56.8 | Other metals | 1 | 0 | 1 |
| T39.8 | Other nonopioid analgesics and antipyretics, not elsewhere classified | 2 | 1 | 3 |
| T39.3 | Other nonsteroidal anti-inflammatory drugs [NSAID] | 3 | 0 | 3 |
| T44.3 | Other parasympatholytics [anticholinergics and antimuscarinics] and spasmolytics, not elsewhere classified | 1 | 1 | 2 |
| T43.3 | Phenothiazine antipsychotics and neuroleptics | 2 | 0 | 2 |
| J69 | Pneumonitis due to food and vomit | 1 | 0 | 1 |
| T43.6 | Psychostimulants with potential for use disorder | 0 | 1 | 1 |
| T43.9 | Psychotropic drug, unspecified | 3 | 0 | 3 |
| T39 | Salicylates | 1 | 0 | 1 |
| T43 | Tricyclic and tetracyclic antidepressants | 3 | 1 | 4 |
|  | **Unspecified/Miscellaneous Drugs** |  | | |
| X44 | Accidental poisoning by and exposure to other and unspecified drugs, medicaments and biological substances | 46 | 48 | 94 |
| X43 | Accidental poisoning by and exposure to other drugs acting on the autonomic nervous system | 3 | 7 | 10 |
| X64 | Intentional self-poisoning by and exposure to other and unspecified drugs, medicaments and biological substances | 27 | 34 | 61 |
| X63 | Intentional self-poisoning by and exposure to other drugs acting on the autonomic nervous system | 13 | 10 | 23 |
| F19.2 | Mental and behavioural disorders due to multiple drug use and use of psychoactive substances, dependence syndrome | 1 | 0 | 1 |
| F19.9 | Mental and behavioural disorders due to multiple drug use and use of psychoactive substances, unspecified mental and behavioural disorder | 1 | 0 | 1 |
| F11.2 | Mental and behavioural disorders due to use of opioids, dependence syndrome | 0 | 1 | 1 |
| F50.9 | Other and unspecified drugs, medicaments and biological substances | 4 | 5 | 9 |
|  | **Diseases/Conditions** |  | | |
| f43 | Acute stress reaction | 6 | 7 | 13 |
| R40.2 | Coma | 0 | 1 | 1 |
| F05.0 | Delirium not superimposed on dementia, so described | 0 | 1 | 1 |
| F32.9 | Depressive episode, unspecified | 0 | 5 | 5 |
| F99 | Mental disorder, not otherwise specified | 0 | 1 | 1 |
| Z03.8 | Observation for other suspected diseases and conditions | 1 | 0 | 1 |
| Z60.9 | Observation for suspected disease or condition, unspecified | 1 | 1 | 2 |
| Z03.2 | Observation for suspected mental and behavioural disorders | 0 | 1 | 1 |
| S51.9 | Open wound of forearm, part unspecified | 0 | 1 | 1 |
| Z91.5 | Personal history of self-harm | 1 | 0 | 1 |
| F60.9 | Personality disorder, unspecified | 0 | 1 | 1 |
| F43.1 | Post-traumatic stress disorder | 1 | 0 | 1 |
| F53.9 | Puerperal mental disorder, unspecified | 0 | 1 | 1 |
| J81 | Pulmonary oedema | 1 | 0 | 1 |
| F20.9 | Schizophrenia, unspecified | 1 | 0 | 1 |
| A48.3 | Toxic shock syndrome | 1 | 3 | 4 |
| N39 | Urinary tract infection, site not specified | 0 | 1 | 1 |
|  | **Other** |  | | |
| Z60.9 | Problem related to social environment, unspecified | 1 | 0 | 1 |
| Z53.9 | Procedure not carried out, unspecified reason | 1 | 2 | 3 |
| T88.7 | Unspecified adverse effect of drug or medicament | 2 | 2 | 4 |
